# Supplementary material for: Fully resolved assembly of Cryptosporidium parvum
Source: Gigascience. 2022 Feb 15;11:giac010. doi: 10.1093/gigascience/giac010 (PMC8848321; doi:10.1093/gigascience/giac010)
Supplement: giac010_GIGA-D-21-00321_Original_Submission [file giac010_giga-d-21-00321_original_submission.pdf]

GigaScience

Fully resolved assembly of Cryptosporidium parvum

--Manuscript Draft--

**Manuscript Number:**	GIGA-D-21-00321
**Full Title:**	Fully resolved assembly of Cryptosporidium parvum
**Article Type:**	Data Note
**Funding Information:**	National Institute of Allergy and Infectious Diseases (1U19AI144297) — Dr Joseph F. Petrosino
**Abstract:**	Background<br>Cryptosporidium parvum is an apicomplexan parasite commonly found across many species with a global infection prevalence of 7.6%. As such, it is important to understand the diversity and genomic makeup of this prevalent parasite to prohibit further spread and to fight infection. The general basis of every genomic study is a high quality reference genome that has continuity and completeness and is of high quality, thus enabling comprehensive comparative studies.<br>Findings<br>Here, we provide a highly accurate and complete reference genome of Cryptosporidium parvum. The assembly is based on Oxford Nanopore reads and was improved using Illumina reads for error correction. We also outlined how to evaluate and choose from different assembly methods based on two main approaches that can be applied to other Cryptosporidium species. The assembly encompasses 8 chromosomes and includes 13 telomeres that were resolved. Overall, the assembly shows a high completion rate with 98.4% single copy BUSCO genes. The consensus accuracy of the established reference genome was further validated by sequence alignment of established genetic markers for C . parvum .<br><br>Conclusions<br>This high quality reference genome of a zoonotic IIa A17G2R1 C. parvum subtype isolate provides the basis for subsequent studies and comparative genomic studies across the Cryptosporidium clade. Thus will enable improved understanding of diversity, functional and thus association studies.
**Corresponding Author:**	Fritz J Sedlazeck<br>Baylor College of Medicine<br>Houston, UNITED STATES
**Corresponding Author Secondary Information:**	
**Corresponding Author's Institution:**	Baylor College of Medicine
**Corresponding Author's Secondary Institution:**	
**First Author:**	Vipin K Menon
**First Author Secondary Information:**	
**Order of Authors:**	Vipin K Menon
	Pablo C Okhuysen
	Cynthia Chappell
	Medhat Mahmoud
	Medhat Mahmoud
	Qingchang Meng
	Harsha Doddapaneni
	Yi Han

Powered by Editorial Manager® and ProduXion Manager® from Aries Systems Corporation

	Vanesa Vee
	Sejal Salvi
	Sravya Bhamidipati
	Kavya Kottapalli
	George Weissenberger
	Hua Shen
	Ginger A. Metcalf
	Matthew C. Ross
	Kristi L. Hoffman
	Sara Javornik Cregeen
	Donna M. Muzny
	Richard A. Gibbs
	Joseph F. Petrosino
	Fritz J Sedlazeck
**Order of Authors Secondary Information:**	
**Additional Information:**	
**Question**	**Response**
Are you submitting this manuscript to a special series or article collection?	No
**Experimental design and statistics**<br><br>Full details of the experimental design and statistical methods used should be given in the Methods section, as detailed in our Minimum Standards Reporting Checklist. Information essential to interpreting the data presented should be made available in the figure legends.<br><br>Have you included all the information requested in your manuscript?	Yes
**Resources**<br><br>A description of all resources used, including antibodies, cell lines, animals and software tools, with enough information to allow them to be uniquely identified, should be included in the Methods section. Authors are strongly encouraged to cite Research Resource Identifiers (RRIDs) for antibodies, model organisms and tools, where possible.	Yes

Have you included the information requested as detailed in our Minimum Standards Reporting Checklist?	
**Availability of data and materials**<br><br>All datasets and code on which the conclusions of the paper rely must be either included in your submission or deposited in publicly available repositories (where available and ethically appropriate), referencing such data using a unique identifier in the references and in the "Availability of Data and Materials" section of your manuscript.<br><br>Have you have met the above requirement as detailed in our Minimum Standards Reporting Checklist?	Yes

Manuscript Click here to access/download;Manuscript;manuscript.docx

Fully resolved assembly of *Cryptosporidium parvum*

Vipin K. Menon[1,*], Pablo C. Okhuysen[2], Cynthia Chappell[3], Medhat Mahmoud[1], Medhat Mahmoud[1], Qingchang Meng[1], Harsha Doddapaneni[1],Vanesa Vee[1], Yi Han[1], Sejal Salvi[1], Sravya Bhamidipati[1], Kavya Kottapalli[1], George Weissenberger[1], Hua Shen[1], Matthew C. Ross[4], Kristi L. Hoffman[4], Sara Javornik Cregeen[4], Donna M. Muzny[1], Ginger A. Metcalf[1], Richard A. Gibbs[1], Joseph F. Petrosino[4], Fritz J. Sedlazeck[1,*]

Corresponding authors*: menon@bcm.edu, fritz.sedlazeck@bcm.edu

1:Human Genome Sequencing Center, Baylor College of Medicine, Houston, Texas, United States of America;
2:Department of Infectious Diseases, The University of Texas MD Anderson Cancer Center, Houston, Texas, United States of America;
3:The University of Texas School of Public Health, Houston, Texas, United States of America;
4:Alkek Center for Metagenomics and Microbiome Research, Department of Molecular Virology and Microbiology, Baylor College of Medicine, Houston, Texas, United States of America

Abstract

Background

Cryptosporidium parvum is an apicomplexan parasite commonly found across many species with a global infection prevalence of 7.6%. As such, it is important to understand the diversity and genomic makeup of this prevalent parasite to prohibit further spread and to fight infection. The general basis of every genomic study is a high quality reference genome that has continuity and completeness and is of high quality, thus enabling comprehensive comparative studies.

Findings

Here, we provide a highly accurate and complete reference genome of *Cryptosporidium* parvum. The assembly is based on Oxford Nanopore reads and was improved using Illumina reads for error correction. We also outlined how to evaluate and choose from different assembly methods based on two main approaches that can be applied to other *Cryptosporidium* species. The assembly encompasses 8 chromosomes and includes 13 telomeres that were resolved. Overall, the assembly shows a high completion rate with

98.4% single copy BUSCO genes. The consensus accuracy of the established reference genome was further validated by sequence alignment of established genetic markers for *C.parvum*.

Conclusions

This high quality reference genome of a zoonotic IIaA17G2R1 *C. parvum* subtype isolate provides the basis for subsequent studies and comparative genomic studies across the *Cryptosporidium* clade. Thus will enable improved understanding of diversity, functional and thus association studies.

Keywords: Assembly, *Cryptosporidium*, nanopore, Assembly comparisons

Introduction

Cryptosporidium spp. is an apicomplexan parasite of public health and veterinary significance, with a recent analysis reporting a global infection prevalence of 7.6% [1]. Historically, limited government and private funding was available to study the epidemiology and molecular dynamics of the organism, but this has recently shifted [2].

Cryptosporidium spp. have been found in 155 species of mammals, including primates [3,4]. Among humans, twenty species of *Cryptosporidium* spp. have been identified [5]. Although the parasite can be transmitted in a variety of ways, the most common method is *via* water (drinking water and recreational water). In the United States, *Cryptosporidium* is the most common cause of waterborne disease in humans [6]. Studies have shown that *Cryptosporidium* is responsible for a large proportion of all cases of moderate-to-severe diarrhea in children under the age of two [7,8]. There is currently no vaccine available, and the only approved drug for the treatment of *Cryptosporidium*-related diarrhea is nitazoxanide (NTZ), which has limited activity in immunocompromised patients.

The inability to grow *Cryptosporidium in vitro* hampered progress in understanding pathogenesis and exploring new treatment modalities. The use of human organoids recapitulates *in vivo* physiology of their original tissues [9][10,11] and the molecular mechanisms and pathways used by *Cryptosporidium* during infection. However, it became apparent that a high quality reference genome is needed to facilitate any genomic or association studies.

C. *parvum* was included in early genome-sequencing projects due to its public health importance and high global prevalence. The first reported complete genome assembly for *C*. *parvum* Iowa II became available in 2004 [12], generated by random shotgun sequencing approach, resulting in roughly 13x genome coverage totaling 9.1 Mb of DNA sequence across all the eight chromosomes. The reference had a reduced coverage

across the genome, with gaps and was not adequate to represent the full breadth of genes present, which could result in misleading interpretations of the isolates being studied. Online repositories such as GenBank, CryptoDB and the Wellcome Trust Sanger Institute FTP servers also have a range of unassembled, unprocessed raw read sequences.

Long-read sequencing technology has advanced to enable read lengths of 15 - 20 Kb (PacBio) and 2 - 3 Mb (Oxford Nanopore (ONT)) with low error rates and is frequently utilized to improve reference genome assembly [5,13–18]. Thus, enabling long continuous assemblies without gaps even across highly repetitive regions [19]. While long-read technologies enable an improved assembly, it is hard to evaluate which *de novo* assembly is the best representation of the sample. The simplest way to rank de novo assemblies currently is by length [19] (N50) or BUSCO [20] comparison. However, this is not a guarantee that chromosomes are well represented or not wrongly arranged. Furthermore, the variety of *de novo* assembly methods (Canu [21], Flye [22], Shasta [23], Falcon [24], etc.) makes it harder to choose the best representation.

In the current study, we have generated a reference genome for *Cryptosporidium parvum* by using long-read sequencing on the ONT PromethION supplemented with short-read data generated on NovaSeq 6000 for error correction (see **Figure 1**). This resulted in a complete reference including all chromosomes and represents a gap-less representation of this important pathogen. Furthermore, it includes almost all telomeric sequences. The assembly is available at PRJNA744539 (GCA_019844115.1). In addition to the novel assembly, we lay out our QC process and assessment of the assembly to optimize not just for length but also assessing the overall structure of the draft assemblies. Following this comparison schema it is easy to choose the most optimal representation. In addition, this schema is applicable for other species as well, from single haploid to more complex organisms like plants or humans.

Results

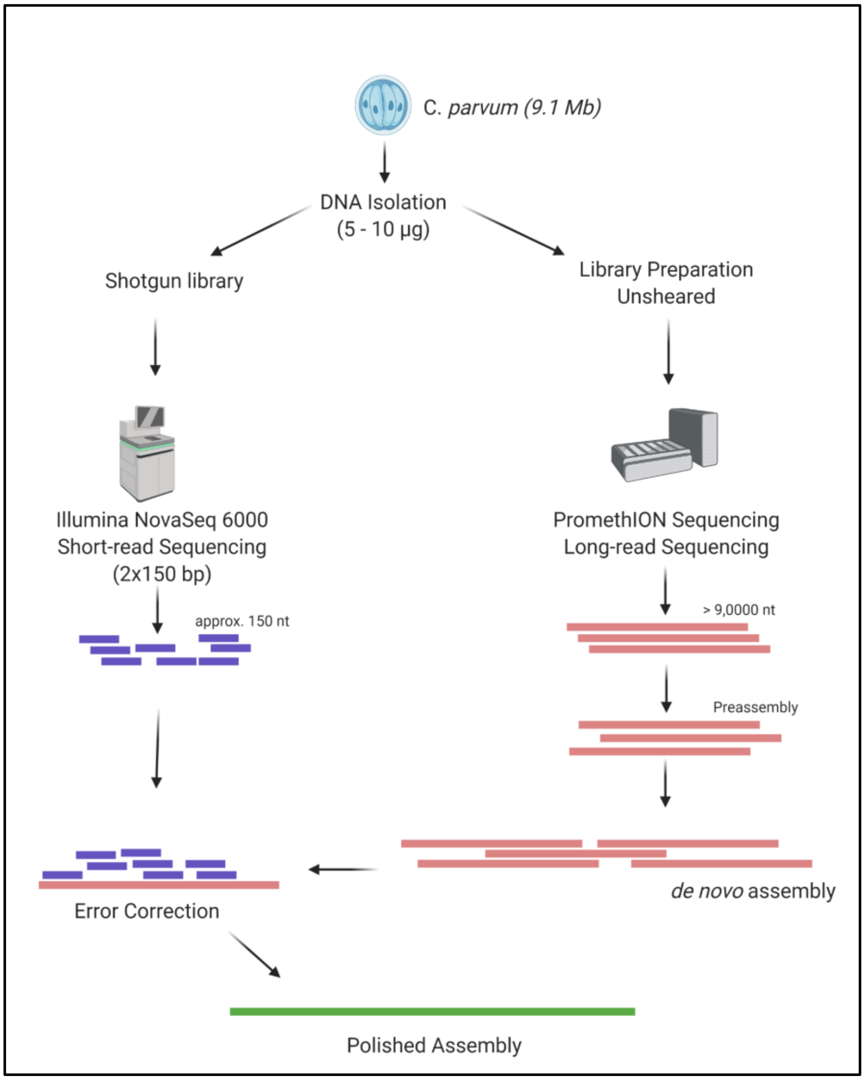

Figure 1: *Workflow for the generation of Cryptosporidium parvum assembly .*

We sequenced the *Cryptosporidium* genome with Oxford Nanopore long-reads (see methods) and obtained a total of ~480Mbp of sequence (**Figure 1**). This is equivalent to 53x coverage for this genome (~9Mbp genome size). **Figure 2** shows overall statistics on read length and coverage. The N50 read length is 15.3 kbp with 10x coverage of reads with ≥30kbp length. Our longest read detected was 808 kbp. In addition, we sequenced the genome using the Illumina NovaSeq 6000 to produce 352x coverage of 150bp paired end reads.

Using these short-reads we ran a genome estimation using GenomeScope [25] to obtain a genome size estimate using a polyploidy of 1. Doing so resulted in an estimate of 9.9Mbp with an 89.24% model fit (see **Supplementary Figure 1**). Inspection of the resulting data shown in the figure highlights that this is a potential overestimation of the genome size itself and thus fits in the realm of the previously reported reference assembly in CryptoDB (GCA_015245375) of ~9.1Mbp.

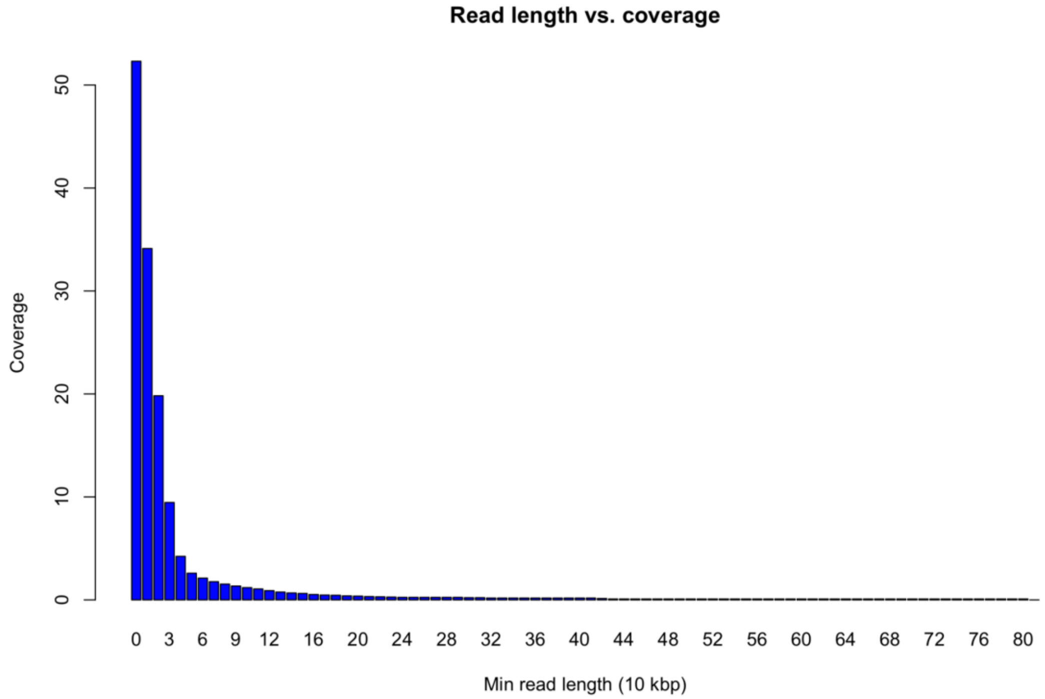

Figure 2: *Read length distribution and cumulative coverage over the Oxford Nanopore. Sequencing. We obtained a total of 53x coverage with long-reads and even 10x coverage with reads larger than 30kbp (x axis). The longest read measured was 808kbp.*

Assembly and comparison of *Cryptosporidium* Assembly

The initial assembly was carried out with only the ONT reads only using Canu [21] (see methods) and resulted in 25 contigs with 8 contigs representing the entire chromosomes. We obtained a total genome length of 9.19Mbp across 8 assembled contigs with an average N50 size of 1.11Mbp (Table 1). The largest contig was 1.4 Mbp. Our assembly shows a NG50 similar to that of the assembly published in 2004 (see **Figure3 A**).

We also ran an assembly with Flye[22] (see methods), which led to a total of 7 contigs. However, one contig was only 62,160 bp long (see **Figure3 B**). Despite this early warning sign, we compared the two assemblies to identify which one represented the *Cryptosporidium* genome best using genome alignments and remapping of short reads.

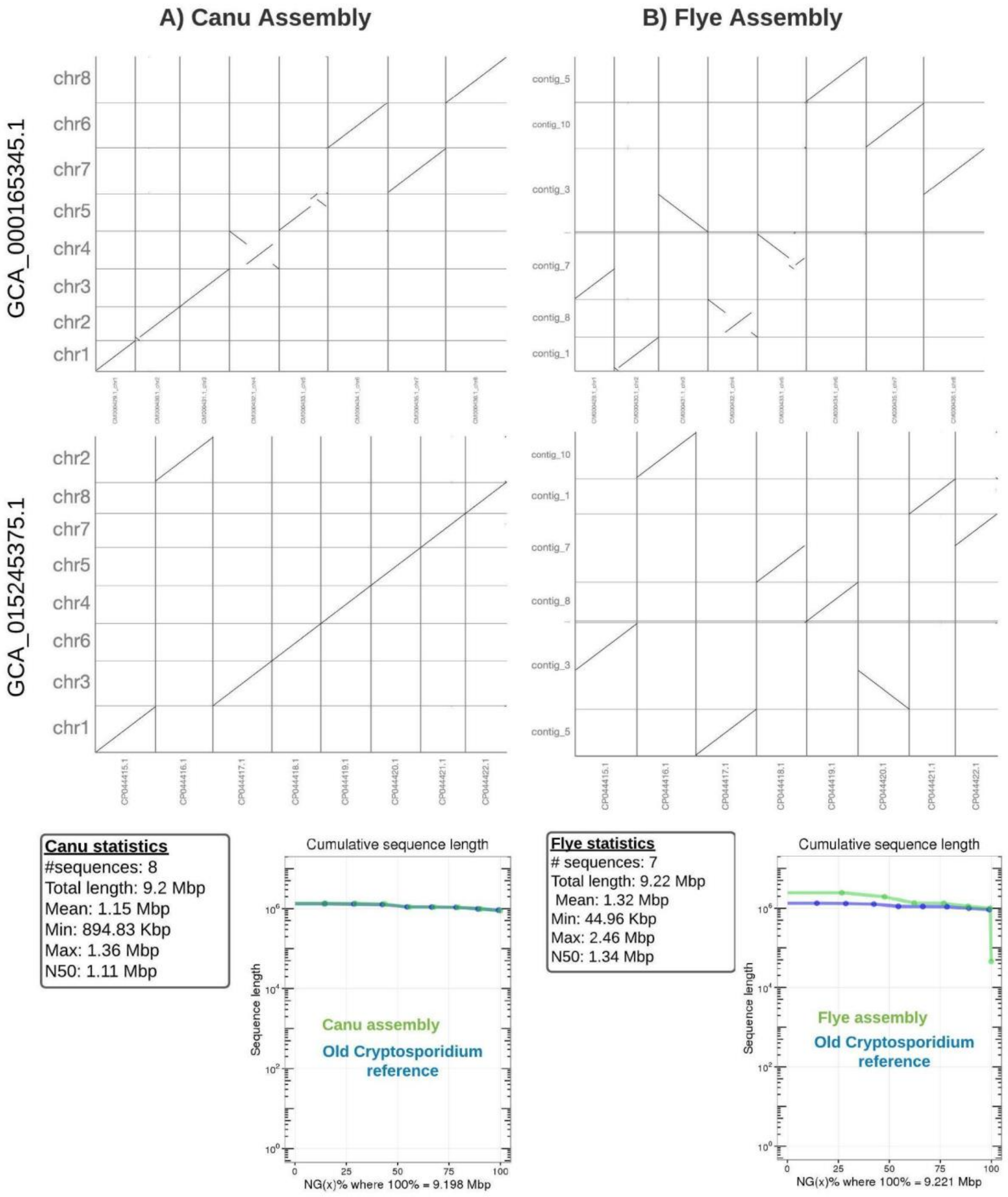

Figure3: *Assembly comparison A) Comparison of the obtained Canu assembly shows a high concordance with the previously published Cryptosporidium assembly (GCA_015245375.1) [26] (dotplots) and agreements in length (bottom). Nevertheless clear assembly differences are visual when comparing it to GCA_000165345.1 [12] B) Comparison of the Flye assembly to the previously published Cryptosporidium assembly (GCA_015245375.1),shows large disagreements. Contig 3 is merged between two different Cryptosporidium chromosomes and one chromosome is missing.*

Also, the length comparison (bottom) shows discrepancies in the beginning highlighting a very short contig in the end (green track). Interestingly GCA_000165345.1 shows structural differences over both assemblies likely indicating errors in the previous reference.

To validate our findings, we first aligned the Canu and Flye assembly to the previously published *Cryptosporidium* reference [3] using nucmer [27](v3.23). The nucmer alignments were filtered by “-l 100 -c 500 -maxmatch” for all assemblies following the suggestions from Assemblytics [28]. Assemblytics was used to study the alignment results that were generated (Figure 3).

The dot plot from a MUMmer alignment analysis indicates that the GCA_015245375.1 [26] and Canu genome assemblies are largely collinear (**Figure 3 A**). All chromosomes show co-linearity to the previously established assembly from *Cryptosporidium*. There are small segments aligned to other chromosomes that upon closer inspection were shown to be telomeric sequences. Thus, these segments did not indicate wrong alignments *per se,* but highlighted their repetitive nature (see below for details on telomere reconstruction). However, when assessing the dot plot of Flye (**Figure 3 B**), we observed larger disagreements compared to GCA_015245375.1. One contig from the Flye assembly was tiny (62 kbp) and was judged to be an artifact, as previously mentioned. More severe, however, was the merger of two *Cryptosporidium* chromosomes into contig3 (**Figure 3B**, second to last row in dotplot). A fusion of two chromosomes from *Cryptosporidium* was also observed on contig_7. Overall, this shows that while we initially missed one contig (7 instead of the expected 8 were reported), one contig was too small (~62kbp) to represent a chromosome, but the missing two chromosomes were merged with other chromosomes within two contigs from Flye. When comparing both of our assemblies (Canu and Flye) to the previously established GCA_000165345.1, we see large structural disagreements on both assembly comparisons (**Figure 3AB**). The differences between GCA_000165345.1 and our *de novo* assemblies are most likely due to structural faults in GCA 000165345.1.

We further conducted a remapping experiment to identify structural disagreements between the Illumina data and the long-read assemblies. We mapped the reads and called Structural Variants (SVs) based on discordant paired end reads (see methods)[29]. We identified a total of 10 potential SVs over the remapping based on the Flye assembly. The majority of events were insertions (4) followed by duplications (3) and breakend (BND) (2). However, on closer inspection only two SVs (the two BND) showed a misassembly with a homozygous alternative genotype. All other 8 SVs showed a minor allele frequency and are likely consequences of mapping artifacts or heterogeneity of the sequenced population. Next, we assessed the Canu assembly, which showed 9 SVs in total. All of the so identified SV showed a low read support and thus also a low probability of being correctly identified. Thus likely originating from mapping artifacts.

This assessment demonstrated that the Canu assembly is the better representation of *Cryptosporidium* compared to Flye for this study. Furthermore, validating the high quality of our assembly.

Establishing *Cryptosporidium* Assembly

Next, we improved the quality of the Canu assembly using the short reads (see methods) over 2 rounds of assembly polishing. After the first round, the number of corrections were reduced to ~20 along the entire genome. After these improvements, we extracted the 8 largest contigs and compared them over a genomic alignment (see methods) to the previously published *Cryptosporidium* reference GCA_015245375.1 [12]. This confirmed that the eight contigs represent the previously published chromosomes, while the other contigs appear to be repeats at the start or end of the contigs. Our assembled eight chromosomes complete 14,669 bp of unresolved sequences (i.e. N). Our assembly also showed a GC content (30.11%) similar to the previous version (30.18%), again attesting to the overall quality.

To further assess the completeness of our assembly, we used Busco [20] with the coccidia_odb10 linkage set (see methods). This analysis confirmed the high quality of our assembly, showing 98.4% complete re-identified genes from a total of 502. All 494 genes had single copies, indicating that the new assembly is error-free. In addition to these single-copy genes, three genes were fragmented, and five genes were missing from the Busco run.

A further comparison to the previous reference genome (GCA_015245375.1) [12] revealed a high consistency with only 4 structural variants (1 insertion, 1 deletion, 1 tandem expansion and 1 tandem contraction) between the two assemblies. This was done based on the genomic alignment and using Assemblytics[28].

	**GCA_000165345.1**	**Current Assembly**
Total sequence length	9,102,324	9,197,619
Total ungapped length	9,087,655	9,197,619
Unresolved sequences	14,669	0
N50	1,104,417	1,108,772
N90	985,969	993,129
L50	4	4
Total number of chromosomes	8	8

Table1: Overall assembly statistics and comparison using Quast of our assembly and the previous established assembly.

Telomere identification

Since our Canu assembly directly reported chromosomes, we next sought to identify telomeric ends on either side of each chromosome (see methods). To search for the telomeres, we identified matching sequences in our assemblies of “TTTAGG” repeats [30] (see methods). We required at least 100 matches within a region towards the start and end of the contigs. Given these conservative thresholds to avoid other repeats, we identified a total of 13 telomeric regions. For the majority of chromosomes (2,3,4,5 and 6) both sides showed telomeric regions. Thus, fully representing the chromosomes from telomere to telomere, including the centromere. On chromosomes 7 and 1, the telomere was only found at the beginning of the chromosome. We only found the telomeric sequence at the end of chromosome 8. We further cross checked the other contigs that were filtered out previously. These highlighted telomeric sequences, but couldn’t be placed automatically to the other chromosomes (chromosome 1, 7 or 8). Overall, the identification of the telomeric sequences on the fast majority of the contigs highlights the overall high quality and continuity of our newly established C. parvum genome. Thus, we deposited the assembly and refer to it under its accession GCA_019844115.1.

Comparative genomics

Cryptosporidium spp. is usually typed and characterized widely by using a small set of genetic markers including *gp60*, COWP, HSP70 and 18S [31]. Most of the genetic marker data available in GenBank are generated from short-read amplification and sequencing by Sanger, thus providing a better resolution, but still contain errors arising from manual curation.

A nucleotide identity matrix was generated post ClustalW alignment carried out using BioEdit V7.2.5, for 18S (Table 2) and *gp60* (Table 3) genetic markers, which were downloaded from GenBank. No gaps or large mismatches between the assembled genome and the genetic markers were observed (see **Supplementary Figure 2 & 3**). There were 0.2% identity mismatches observed in some references, which represent variations observed within the species.

	AF161856.1	AF108864.1	AB513864.1	AF040725.1	MN914085.1	MN914084.1	GCA_019844115.1_18S
AF161856.1		1	0.999	0.999	0.998	0.998	1
AF108864.1	1		0.999	0.999	0.998	0.998	1
AB513864.1	0.999	0.999		0.998	0.998	0.998	0.999
AF040725.1	0.999	0.999	0.998		0.998	0.998	0.999
MN914085.1	0.998	0.998	0.998	0.998		1	0.998
MN914084.1	0.998	0.998	0.998	0.998	1		0.998
GCA_019844115.1_18S	1	1	0.999	0.999	0.998	0.998	

Table 2: Sequence identity matrix of 18S genes from *Cryptosporidium parvum species* showing the sequence identity on 0-1 scale between data from GenBank to the current assembly (GCA_019844115.1_18S).

	MK034695.1	AY048666.1	AY048665.1	AF155624.1	MK034689.1	AF164489.1	AF114166.1	MK034688.1	GCA_019844115.1_gp60
MK034695.1		1	1	1	0.993	0.993	0.993	0.99	0.996
AY048666.1	1		1	1	0.993	0.993	0.993	0.99	0.996
AY048665.1	1	1		1	0.993	0.993	0.993	0.99	0.996
AF155624.1	1	1	1		0.993	0.993	0.993	0.99	0.996
MK034689.1	0.993	0.993	0.993	0.993		1	1	0.996	0.99
AF164489.1	0.993	0.993	0.993	0.993	1		1	0.996	0.99
AF114166.1	0.993	0.993	0.993	0.993	1	1		0.996	0.99
MK034688.1	0.99	0.99	0.99	0.99	0.996	0.996	0.996		0.987
GCA_019844115.1_gp60	0.996	0.996	0.996	0.996	0.99	0.99	0.99	0.987	

Table 3: Sequence identity matrix of *gp60* genes from *Cryptosporidium parvum species* showing the sequence identity on 0-1 scale between data from GenBank to the gp60 region of the current assembly (GCA_0198441151.1_gp60).

Conclusion

The current work highlights how next-generation sequencing, including third-generation long-read sequencing, can be used to generate a high-quality entire genome assembly, complete with centromeric regions and numerous telomeres. The genome assembly generated provides a gapless reference compared to the previously published GCA_000165345.1 [12] and extends into some telomeric regions over GCA_015245375.1 [26]. The latter did improve over GCA_000165345.1, but is a hybrid assembly based on two different subtypes of Cryptosporidium spp. (IIaA17G2R1 and IIaA15G2R1), which might impact further comparison or association studies. In contrast, our study was able to boost the fidelity and robustness of the assembly by focusing on one subtype only, IIaA17G2R1 resulting in a better telomere to telomere assembly representation (GCA_019844115.1). Studies of *Cryptosporidium spp. are* based on genetic markers previously identified for some regions of chromosome 6, and are not able to provide a better understanding of the genetic variation and recombination occurring within the species. Establishing stronger marker genes and perhaps enabling improved recovery of *Cryptosporidium*-specific sequencing reads by mapping to a high-resolution reference genome will enable better understanding of *Cryptosporidium* transmission.

A commonly used approach for *C. parvum* subtyping is based on tandem repeat analysis of *gp60,* a highly polymorphic gene that encodes for an immunodominant glycoprotein 15/40 located on the surface of sporozoites and merozoites belonging to many *Cryptosporidium* species[32]. The current study was done on an isolate propagated in calves by BGF (Bunch Grass Farms, Deary, ID). The vendor originally propagated *C. parvum* IOWA II which belongs to subtype IIaA15G2R1 based on *gp60* sequencing. This strain has now been replaced with a closely related local isolate belonging to the IIaA17G2R1 subtype and was used here. In our work, this isolate is referred to as *C. parvum* (GCA_019844115.1). It is unclear if the IIaA17G2R1 evolved from IOWA II possibly from recombination with another local isolate or if it represents a

distinct isolate on its own. Nevertheless, the assembly done here to our knowledge represents the first IIaA17G2R1 subtype isolate for which long read sequencing has been performed. *C. parvum* belonging to the IIaA17G2R1 subtype have been identified in farms in various regions of the world[33–35], was the second most common genotype identified in human cases in a recent study done in Canada[36] and is responsible for causing foodborne outbreaks in the US[37,38]

Published studies have shown the presence of contingency genes in *Cryptosporidium* spp., which are responsible for surmounting challenges from the host and are subject to spontaneous mutation rates [39–41]. The majority of these genes are located in the telomere regions of the chromosomes, which are prime sites that evolve and mediate host-parasite interactions [30,42]. In the current assembly, we are able to resolve 13 of the estimated 16 telomeres. The capacity to resolve telomeres and subtelomeres across chromosomes in *Cryptosporidium* spp. allows researchers to better comprehend the organism's adaptation to a variety of environmental and host settings.

We utilized two *de novo* assembly approaches here to obtain a better representation for *Cryptosporidium* spp. We demonstrated two methods for validating these two assemblies throughout the paper. First we compared the assemblies from Flye and Canu to pre existing assemblies from *Cryptosporidium* spp, which are from different subtypes. Here we could identify certain structural differences already. Nevertheless, the detection of structural variations (SVs) itself proved very helpful in the decision of which assembly best represents the species at hand[19]. This was only possible over having orthogonal sequenced Illumina reads. Other studies might choose a different strategy like utilizing HiC directly, which would also enable a better scaffolding [43]. For *Cryptosporidium* spp this was not necessary as the genome is of relatively small size (~9Mbp) and we knew from previous studies that it incompasses 8 chromosomes. The analysis of Busco is also a very important indication of quality (ie. completeness and redundancy). Nevertheless, it doesn't indicate wrong rearrangements that happened on the Flye assembly. Only by comparing to closely related reference genomes and/or orthologous data sets (e.g. Illumina short reads) can these types of misassemblies be readily identified.

The final *Cryptosporidium* spp. assembly will be a helpful resource to study this important pathogen further and investigate its complexity during growth and development *in vitro*, and will also serve as a reference for the study of genetic diversity among different isolates. Furthermore, we hope it also facilitates the work of others carrying out translational research that focuses on characterizing the virulence, pathogenicity,host specificity and targets for control via vaccines or effective antiparasitic agents to treat this and other *Cryptosporidium* spp.

Methods

DNA extraction: *Cryptosporidium parvum* oocysts were obtained from Bunchgrass Farm in Deary, ID (Lot #22-20, shed date, 10/2/20) and are propagated from IOWA-1 subtype IIaA15G2R1, which was recently replaced by a local isolate subtype IIaA17G2R1[44]. Purified oocysts (10^8) were washed in PBS and treated with diluted bleach for 10 minutes on ice to allow for sporozoite excystation. Parasites were pelleted, washed in PBS, and DNA was extracted using Ultrapure™ phenol:chloroform:isoamyl alcohol (Thermo Scientific) followed by ethanol precipitation. Glycoblue™ co-precipitant (Thermo Scientific) was used to facilitate visualization of DNA during extraction and purification steps.

ONT Library preparation & sequencing

NEBNext FFPE DNA Repair Mix was used to repair 620ng of genomic DNA, which was then followed by end-repair and dA-tailing with NEBNext Ultra II reagents. The dA-tailed insert molecules were further ligated with an Oxford Nanopore adaptor via ligation kit SQK-LSK110. Purification of the library was carried out with AMPure XP beads (Beckman, Cat# A63880), the final library of 281ng was loaded to one PromethION 24 flow cell (FLO-PRO002) and the sequencing data was collected for 24 hours.

Illumina Library preparation & sequencing

DNA (100 ng) was sheared into fragments of approximately 300-400 bp in a Covaris E210 system (96 well format, Covaris, Inc. Woburn, MA) followed by purification of the fragmented DNA using AMPure XP beads. DNA end repair, 3'-adenylation, ligation to Illumina multiplexing dual-index adaptors, and ligation-mediated PCR (LM-PCR) were all completed using automated processes. The KAPA HiFi polymerase (KAPA Biosystems Inc.) was used for PCR amplification (10 cycles), which is known to amplify high GC and low AT rich regions at greater efficiency. A fragment analyzer (Advanced Analytical Technologies, Inc) electrophoresis system was used for library quantification and size estimation. The libraries were 630 bp (including adaptor and barcode), on average. The library was pooled with other internal samples, with adjustment carried out to yield 3 Gbp of data on a NovaSeq 6000 S4 flow cell.

Genome size estimation

We used Jellyfish (version 2.3.0) to generate a k-mer based histogram of our raw reads in order to estimate the genome size based on our short read data. To obtain this we ran Jellyfish[45,46] with " jellyfish count -C -m 21 -s 1000000000 -t 10" and subsequently the "histo" module with default parameters. The obtained histogram was loaded into GenomeScope[45] given the appropriate parameter (k-mer size of 21) and haploid genome. GenomeScope provided the overall statistics across the short reads.

Assembly evaluation

We aligned the assembly of Canu (version 2.0)[21] and Flye (version 2.8.1-b1676) [22] with the two *Cryptosporidium* assemblies GCA_000165345.1 and GCA_015245375.1 using nucmer (version 3.1) -maxmatch -l 100 -c 500 [27]. Next, the delta files were evaluated with Assemblytics[28] (version 1.2.1) (assemblytics.com) using the dotplot function. In addition, we mapped the short Illumina reads using bwa mem[47] (0.7.17-r1188) with default parameters. Subsequently, we identified Structural Variants using Manta[48] (v1.6.0) and assessed the VCF file manually.

Assembly and polishing

We utilized Canu [21] (v2.0) for the assembly, which was based only on Nanopore pass data and a genome size estimate of 9Mbp. On the Nanopore pass reads, we also ran the assembly using Flye [22] (version 2.8.1-b1676) with the default parameters. Subsequently, we aligned the short reads using bwa-mem (version 0.7.17-r1188) with -M -t 10 parameters. Samtools[49] (v1.9) was used to compress and sort the alignments. The so generated alignment was used by Pilon [50] (v 1.24) with the parameters "--fix bases "by correcting one chromosome after another of the raw assembly. This process was repeated two times achieving a high concordance of the reads and the long-read assembly at the 2nd polishing step.

BUSCO assessment

We ran BUSCO [20] (v5.1.3) to assess the completeness of our assembly using default parameter and --auto-lineage, but lineage was detected to coccidia_odb10 (Creation date: 2020-08-05, number of genomes: 20, number of BUSCOs: 502). We reported the stats from the output summary file from BUSCO in this manuscript.

Telomere Identification

We used the sequence "TTTAGGTTTAGGTTTAGG" to identify telomeric sequences at the start and end of every contig from our assembly. To do so we used Bowtie[51] (version 1.2.3) to align the telomeric sequence back to the assembly with -a parameter. Subsequently we counted the matches across regions using a custom script. In short, we used 10kbp windows to count the number of reported hits, align the genome and compare the locations with the expected start/end locations. The identified regions were filtered for at least 100 hits to guarantee a robust match. This way, we counted the number of times each chromosome was listed.

Regional comparison

Two genetic markers 18S and gp60 were used to determine any significant gaps or mismatches against available GenBank genomes for *C.parvum*. The 18S and *gp60* coding regions downloaded from GenBank were aligned using ClustalW [52](BioEdit

V7.2.5) against the current assembly. For each gene, a similarity identity matrix table was created. Further analysis of the G60 gene sequence for tandem repeats to determine subtype designation was done following the methods of Alves et. al. [53]

Additional Files

Supplemental Figure 1. Genomescope estimation of genome size
Supplemental Figure 2. NG50 comparison of the previous reference
Supplemental Figure 3. ClustalW alignment of the 18S coding sequence with the assembly.
Supplemental Figure 4. ClustalW alignment of the *gp60* coding sequence with the assembly.

Competing Interests
The corresponding author of the paper has presented at both ONT and PacBio sponsored conferences.

Funding
This work was supported by the National Institute of Allergy and Infectious Diseases (Grant#1U19AI144297).

Authors' Contributions
F.J.S and V.K.M : Conceptualization, Analysis and Writing-Original Draft Preparation
C.C and G.A.M : Conceptualization and Writing-Review & Editing
P.C.O : Conceptualization, Resources and Writing-Review & Editing
H.D.; Q.M. and D.M.M. : Conceptualization, Writing-Review & Editing
S.S.; S.B.; K.K.; G. W.; H.S.; V.V.; Y.H. : Methodology, Investigation
M.C.R.; K.L.H.; S.J.C. : Conceptualization
M.M; M.M..: Analysis
R.A.G.; J.F.P. : Conceptualization, Funding Acquisition

References:

1. Dong S, Yang Y, Wang Y, Yang D, Yang Y, Shi Y, et al.. Prevalence of Cryptosporidium Infection in the Global Population: A Systematic Review and Meta-analysis. *Acta Parasitol*. 65:882–92020;

2. Head MG, Brown RJ, Newell M-L, Scott JAG, Batchelor J, Atun R. The allocation of USdollar;105 billion in global funding from G20 countries for infectious disease research between 2000 and 2017: a content analysis of investments. *Lancet Glob Health*. 8:e1295–3042020;

3. Fayer R, Morgan U, Upton SJ. Epidemiology of Cryptosporidium: transmission, detection and identification. *Int J Parasitol*. 30:1305–222000;

4. Fayer R. Cryptosporidium: a water-borne zoonotic parasite. *Vet Parasitol*. 126:37–562004;

5. Xiao L, Feng Y. Molecular epidemiologic tools for waterborne pathogens Cryptosporidium spp. and Giardia duodenalis. *Food Waterborne Parasitol*. 8-9:14–322017;

6. : Parasites - Cryptosporidium (also known as “Crypto”). https://www.cdc.gov/parasites/crypto/index.html (2019). Accessed 2021 May 20.

7. Platts-Mills JA, Babji S, Bodhidatta L, Gratz J, Haque R, Havt A, et al.. Pathogen-specific burdens of community diarrhoea in developing countries: a multisite birth cohort study (MAL-ED). *Lancet Glob Health*. 3:e564–752015;

8. Kotloff KL, Nataro JP, Blackwelder WC, Nasrin D, Farag TH, Panchalingam S, et al.. Burden and aetiology of diarrhoeal disease in infants and young children in developing countries (the Global Enteric Multicenter Study, GEMS): a prospective, case-control study. *Lancet*. 382:209–222013;

9. Heo I, Dutta D, Schaefer DA, Iakobachvili N, Artegiani B, Sachs N, et al.. Modelling Cryptosporidium infection in human small intestinal and lung organoids. *Nat Microbiol*. 3:814–232018;

10. Cardenas D, Bhalchandra S, Lamisere H, Chen Y, Zeng X-L, Ramani S, et al.. Two- and Three-Dimensional Bioengineered Human Intestinal Tissue Models for Cryptosporidium. *Methods Mol Biol*. 2052:373–4022020;

11. Vinayak S, Pawlowic MC, Sateriale A, Brooks CF, Studstill CJ, Bar-Peled Y, et al.. Genetic modification of the diarrhoeal pathogen Cryptosporidium parvum. *Nature*. 523:477–802015;

12. Abrahamsen MS, Templeton TJ, Enomoto S, Abrahante JE, Zhu G, Lancto CA, et al.. Complete genome sequence of the apicomplexan, Cryptosporidium parvum. *Science*. 304:441–52004;

13. Dong L, Wang X, Guo H, Zhang X, Zhang M, Tang W. Chromosome-level genome assembly of the endangered humphead wrasse Cheilinus undulatus: Insight into the expansion of opsin genes in fishes. *Mol Ecol Resour*. 2021; doi: 10.1111/1755-0998.13429.

14. Brancaccio RN, Robitaille A, Dutta S, Rollison DE, Tommasino M, Gheit T. MinION nanopore sequencing and assembly of a complete human papillomavirus genome. *J Virol Methods*. 294:1141802021;

15. Espiritu HM, Mamuad LL, Jin S-J, Kim S-H, Lee S-S, Cho Y-I. High quality genome sequence of Treponema phagedenis KS1 isolated from bovine digital dermatitis. *Hanguk Tongmul Chawon Kwahakhoe Chi*. 62:948–512020;

16. Cuscó A, Pérez D, Viñes J, Fàbregas N, Francino O. Long-read metagenomics retrieves complete single-contig bacterial genomes from canine feces. *BMC Genomics*. 22:3302021;

17. Sun F, Sun S, Ye W, Duan C, Li B, Shan W, et al.. Genome Sequence Data of three formae speciales of Phytophthora vignae Causing Phytophthora Stem Rot on different Vigna species. *Plant Dis*. 2021; doi: 10.1094/PDIS-11-20-2546-A.

18. De Coster W, Weissensteiner MH, Sedlazeck FJ. Towards population-scale long-read sequencing. *Nat Rev Genet*. 2021; doi: 10.1038/s41576-021-00367-3.

19. Sedlazeck FJ, Lee H, Darby CA, Schatz MC. Piercing the dark matter: bioinformatics of long-range sequencing and mapping. *Nat Rev Genet*. 19:329–462018;

20. Simão FA, Waterhouse RM, Ioannidis P, Kriventseva EV, Zdobnov EM. BUSCO: assessing genome assembly and annotation completeness with single-copy orthologs. *Bioinformatics*. 31:3210–22015;

21. Koren S, Walenz BP, Berlin K, Miller JR, Bergman NH, Phillippy AM. Canu: scalable and accurate long-read assembly via adaptive k-mer weighting and repeat separation. *Genome Res*. 27:722–362017;

22. Kolmogorov M, Yuan J, Lin Y, Pevzner PA. Assembly of long, error-prone reads using repeat graphs. *Nat Biotechnol*. 37:540–62019;

23. Shafin K, Pesout T, Lorig-Roach R, Haukness M, Olsen HE, Bosworth C, et al.. Nanopore sequencing and the Shasta toolkit enable efficient de novo assembly of eleven human genomes. *Nat Biotechnol*. 38:1044–532020;

24. Chin C-S, Peluso P, Sedlazeck FJ, Nattestad M, Concepcion GT, Clum A, et al.. Phased diploid genome assembly with single-molecule real-time sequencing. *Nat Methods*. 13:1050–42016;

25. Vurture GW, Sedlazeck FJ, Nattestad M, Underwood CJ, Fang H, Gurtowski J, et al.. GenomeScope: fast reference-free genome profiling from short reads. *Bioinformatics*. 33:2202–42017;

26. Baptista RP, Li Y, Sateriale A, Sanders MJ, Brooks KL, Tracey A, et al.. Long-read assembly and comparative evidence-based reanalysis of Cryptosporidium genome sequences reveal new biological insights. bioRxiv. bioRxiv;

27. Kurtz S, Phillippy A, Delcher AL, Smoot M, Shumway M, Antonescu C, et al.. Versatile and open software for comparing large genomes. *Genome Biol*. 5:R122004;

28. Nattestad M, Schatz MC. Assemblytics: a web analytics tool for the detection of variants from an assembly. *Bioinformatics*. 32:3021–32016;

29. Mahmoud M, Gobet N, Cruz-Dávalos DI, Mounier N, Dessimoz C, Sedlazeck FJ. Structural variant calling: the long and the short of it. Genome Biology.

30. Liu C, Schroeder AA, Kapur V, Abrahamsen MS. Telomeric sequences of Cryptosporidium parvum. *Mol Biochem Parasitol*. 94:291–61998;

31. Widmer G, Sullivan S. Genomics and population biology of Cryptosporidium species. *Parasite Immunol*. 34:61–712012;

32. Strong WB, Gut J, Nelson RG. Cloning and sequence analysis of a highly polymorphic Cryptosporidium parvum gene encoding a 60-kilodalton glycoprotein and characterization of its 15- and 45-kilodalton zoite surface antigen products. *Infect Immun*. 68:4117–342000;

33. Mi R, Wang X, Huang Y, Zhou P, Liu Y, Chen Y, et al.. Prevalence and molecular characterization of Cryptosporidium in goats across four provincial level areas in China. *PLoS One*. 9:e1111642014;

34. Kaupke A, Rzeżutka A. Emergence of novel subtypes of Cryptosporidium parvum in calves in Poland. *Parasitol Res*. 114:4709–162015;

35. Caffarena RD, Meireles MV, Carrasco-Letelier L, Picasso-Risso C, Santana BN, Riet-Correa F, et al.. Dairy Calves in Uruguay Are Reservoirs of Zoonotic Subtypes of and Pose a Potential Risk of Surface Water Contamination. *Front Vet Sci*. 7:5622020;

36. Guy RA, Yanta CA, Muchaal PK, Rankin MA, Thivierge K, Lau R, et al.. Molecular characterization of Cryptosporidium isolates from humans in Ontario, Canada. *Parasit Vectors*. 14:692021;

37. Blackburn BG, Mazurek JM, Hlavsa M, Park J, Tillapaw M, Parrish M, et al.. Cryptosporidiosis associated with ozonated apple cider. *Emerg Infect Dis*. 12:684–62006;

38. Centers for Disease Control and Prevention (CDC). Cryptosporidiosis outbreak at a summer camp--North Carolina, 2009. *MMWR Morb Mortal Wkly Rep*. 60:918–222011;

39. Bouzid M, Tyler KM, Christen R, Chalmers RM, Elwin K, Hunter PR. Multi-locus analysis of human infective Cryptosporidium species and subtypes using ten novel genetic loci. *BMC Microbiol*. 10:2132010;

40. Widmer G, Lee Y, Hunt P, Martinelli A, Tolkoff M, Bodi K. Comparative genome analysis of two Cryptosporidium parvum isolates with different host range. *Infect Genet Evol*. 12:1213–212012;

41. Moxon ER, Lenski RE, Rainey PB. Adaptive evolution of highly mutable loci in pathogenic bacteria. *Perspect Biol Med*. 42:154–51998;

42. Bouzid M, Hunter PR, Chalmers RM, Tyler KM. Cryptosporidium pathogenicity and virulence. *Clin Microbiol Rev*. 26:115–342013;

43. Kadota M, Nishimura O, Miura H, Tanaka K, Hiratani I, Kuraku S. Multifaceted Hi-C benchmarking: what makes a difference in chromosome-scale genome scaffolding? *Gigascience*. 2020; doi: 10.1093/gigascience/giz158.

44. Zhang H, Zhu G. High-Throughput Screening of Drugs Against the Growth of Cryptosporidium parvum In Vitro by qRT-PCR. *Methods Mol Biol*. 2052:319–342020;

45. Ranallo-Benavidez TR, Jaron KS, Schatz MC. GenomeScope 2.0 and Smudgeplot for reference-free profiling of polyploid genomes. *Nat Commun*. 11:14322020;

46. Marçais G, Kingsford C. A fast, lock-free approach for efficient parallel counting of occurrences of k-mers. *Bioinformatics*. 27:764–702011;

47. Li H. Aligning sequence reads, clone sequences and assembly contigs with BWA-MEM. arXiv [q-bio.GN].

48. Chen X, Schulz-Trieglaff O, Shaw R, Barnes B, Schlesinger F, Källberg M, et al.. Manta: rapid detection of structural variants and indels for germline and cancer sequencing applications. *Bioinformatics*. 32:1220–22016;

49. Li H, Handsaker B, Wysoker A, Fennell T, Ruan J, Homer N, et al.. The Sequence Alignment/Map format and SAMtools. Bioinformatics.

50. Walker BJ, Abeel T, Shea T, Priest M, Abouelliel A, Sakthikumar S, et al.. Pilon: an integrated tool for comprehensive microbial variant detection and genome assembly improvement. *PLoS One*. 9:e1129632014;

51. Langmead B, Salzberg SL. Fast gapped-read alignment with Bowtie 2. *Nat Methods*. 9:357–92012;

52. Thompson JD, Higgins DG, Gibson TJ. CLUSTAL W: improving the sensitivity of progressive multiple sequence alignment through sequence weighting, position-specific gap penalties and weight matrix choice. *Nucleic Acids Res*. 22:4673–801994;

53. Alves M, Ribeiro AM, Neto C, Ferreira E, Benoliel MJ, Antunes F, et al.. Distribution of Cryptosporidium species and subtypes in water samples in Portugal: a preliminary study. *J Eukaryot Microbiol*. 53 Suppl 1:S24–52006;

Click here to access/download
Supplementary Material
supplement.pdf

Personal Cover Click here to access/download;Personal Cover;Cover_Letter.pdf

October 8th, 2021

Baylor College of Medicine
Human Genome Sequencing Center

One Baylor Plaza
Houston, Texas 77030-3498

TEL: (713)798-6539
FAX: (713)798-5741

Dear Scott Edmunds,

Please find enclosed a copy of the manuscript "Fully resolved assembly of Cryptosporidium parvum", which we submit for consideration to *Gigascience*. We had previously communicated about this manuscript and an extension of it (Mail July 20th).

In our manuscript, we present a telomere to telomere assembly of the important pathogen *Cryptosporidium parvum* that by far exceeds the previous established de novo assembly. As previously suggested, we extended the paper with a section about the QC of the assembly and how to choose one assembly over the other. We further extended the introduction and the abstract to highlight this section.

As you know, *Cryptosporidium parvum* are apicomplexan parasites that are affecting not only humans but a range of species. In humans *Cryptosporidium parvum* lead to diarrhea, anorexia, nausea/vomiting and abdominal pain. In cases of immune compromised patient this can also often lead to death. In this manuscript, we have highlighted our advancement for a much improved assembly reporting a gapless version with 13 telomers completely resolved. The assembly is based on an Oxford Nanopore run combined with an Illumina library for improving the post assembly quality. The assembly has a 98.4% single copy Busco score. We used the assembly for a comparative study of diversity and to show its high quality compared to other *Cryptosporidium* de novo assemblies.

We further suggest potential reviewers based on their experience working on genomic data and *Cryptosporidium*: Giovanni Widmer, PhD, Tufts University School of Veterinary Medicine (Giovanni.widmer@tufts.edu); Rachel Chalmers, PhD, Director, Cryptosporidium Reference Laboratory (Rachel.chalmers@Wales.nhs.uk); Una Ryan, PhD, School of Veterinary and Life Sciences, Murdoch University, Murdoch, Western Australia (Una.Ryan@murdoch.edu.au); Sitara SR Ajjampur, PhD The Welcome Trust Research Laboratory, Division of Gastrointestinal Sciences India (sitararao@cmcvellore.ac.in); Fiona brinkman, PhD School of Computing Science and Faculty of Health Sciences Simon Fraser University (brinkman@sfu.ca); Holly Bik, PhD Department of Marine Sciences, University of Georgia (hbik@uga.edu)

Warm Regards,

Fritz J. Sedlazeck, Ph.D.

Associate Professor,
Human Genome Sequencing Center,
Baylor College of Medicine,
Houston, TX, 77030
