# Supplementary material for: Fully resolved assembly of Cryptosporidium parvum
Source: Gigascience. 2022 Feb 15;11:giac010. doi: 10.1093/gigascience/giac010 (PMC8848321; doi:10.1093/gigascience/giac010)
Supplement: giac010_Supplemental_File [file giac010_supplemental_file.docx]

# Supplement


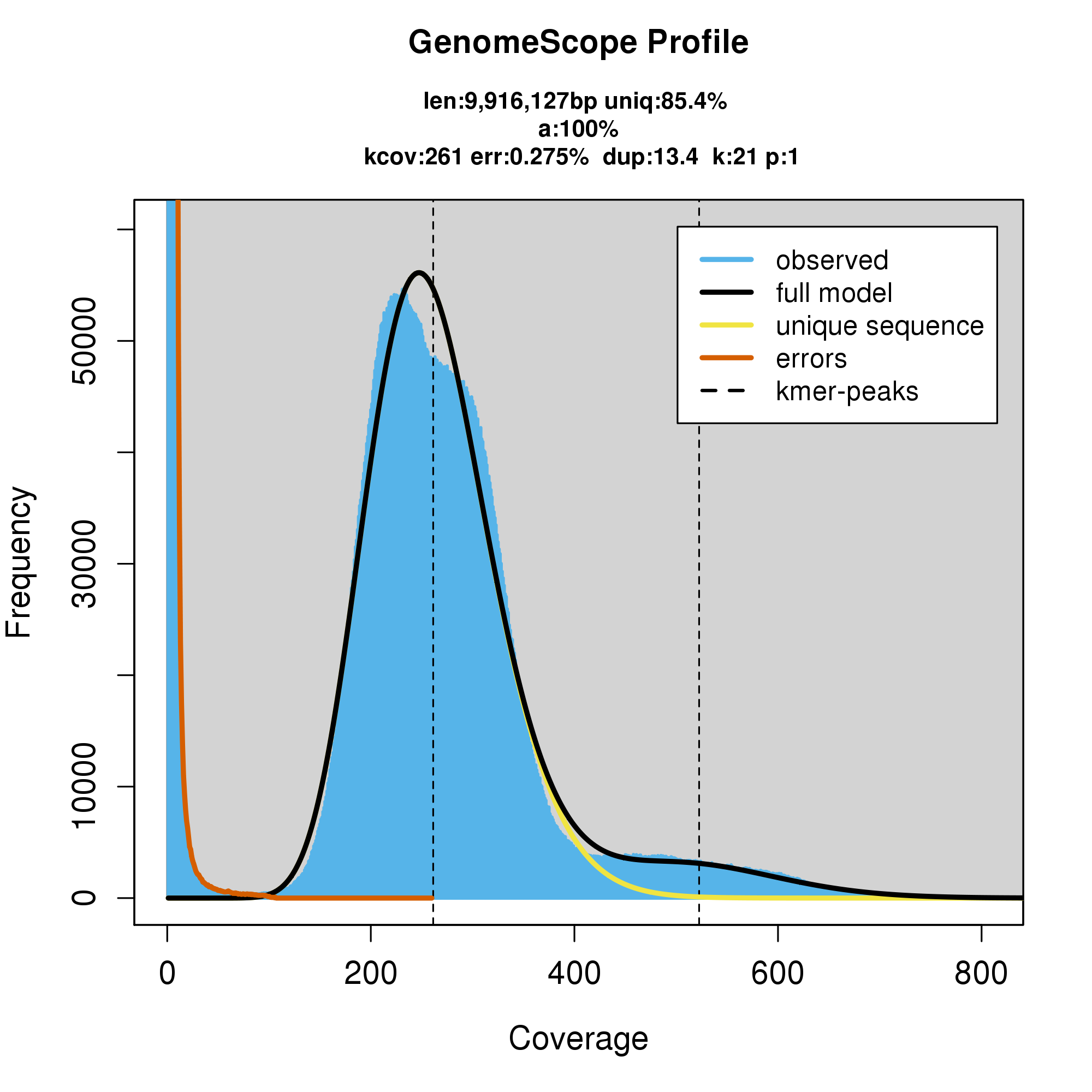


**Supplementary Figure 1:** Genomescope estimation of genome size. The model predicted a genome size of 9.9 Mbp, but as one can see the model fit is only 85.4% not well representing the tip of the variant (black) peak. Thus likely the estimation of 9.9Mbp is a maximum size.


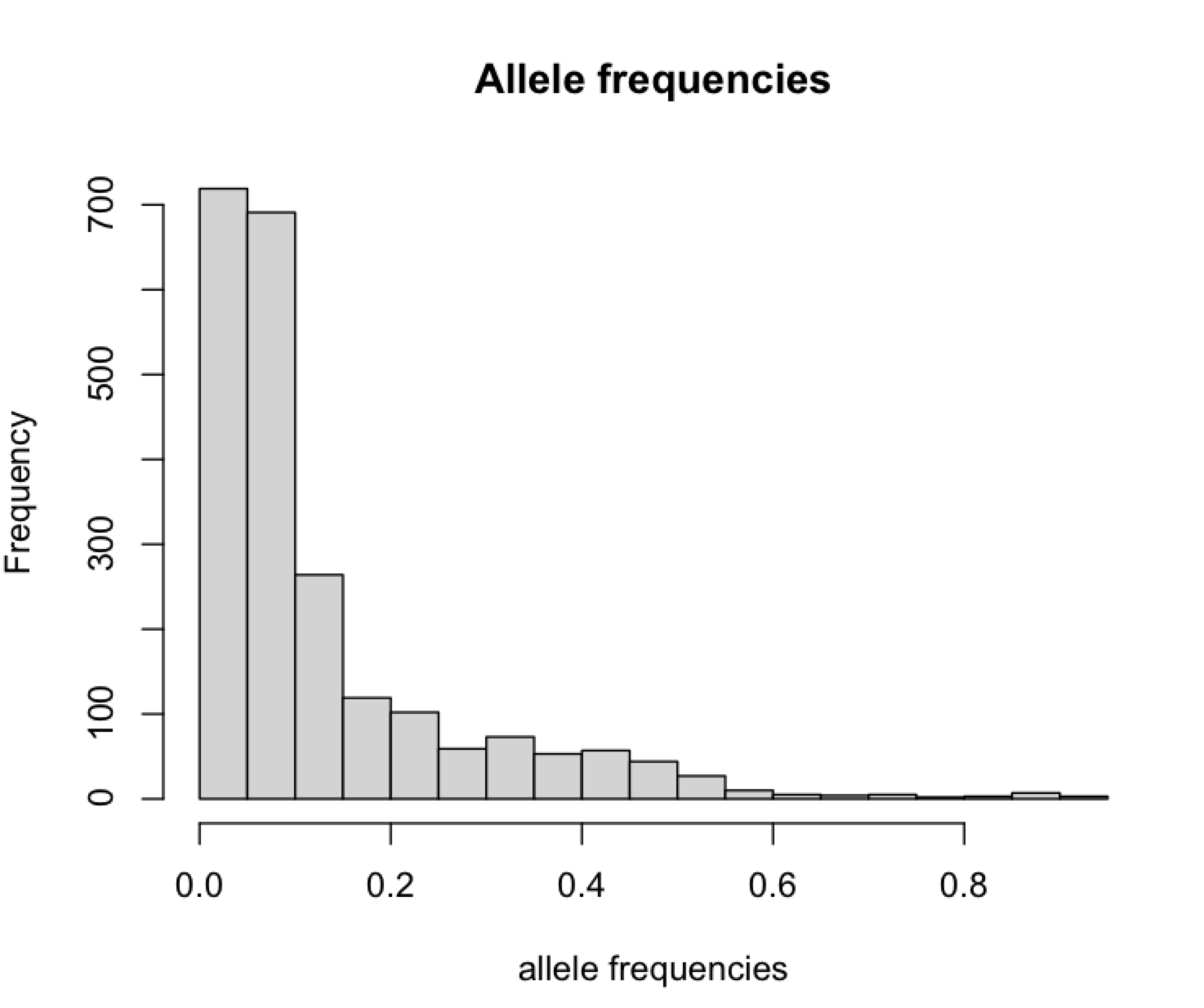


**Supplement Figure 2:** Allele frequency spectrum of remapping Illumina data to our newly established assembly GCA_019844115.1 showing high agreement and little variability across low frequency variants.


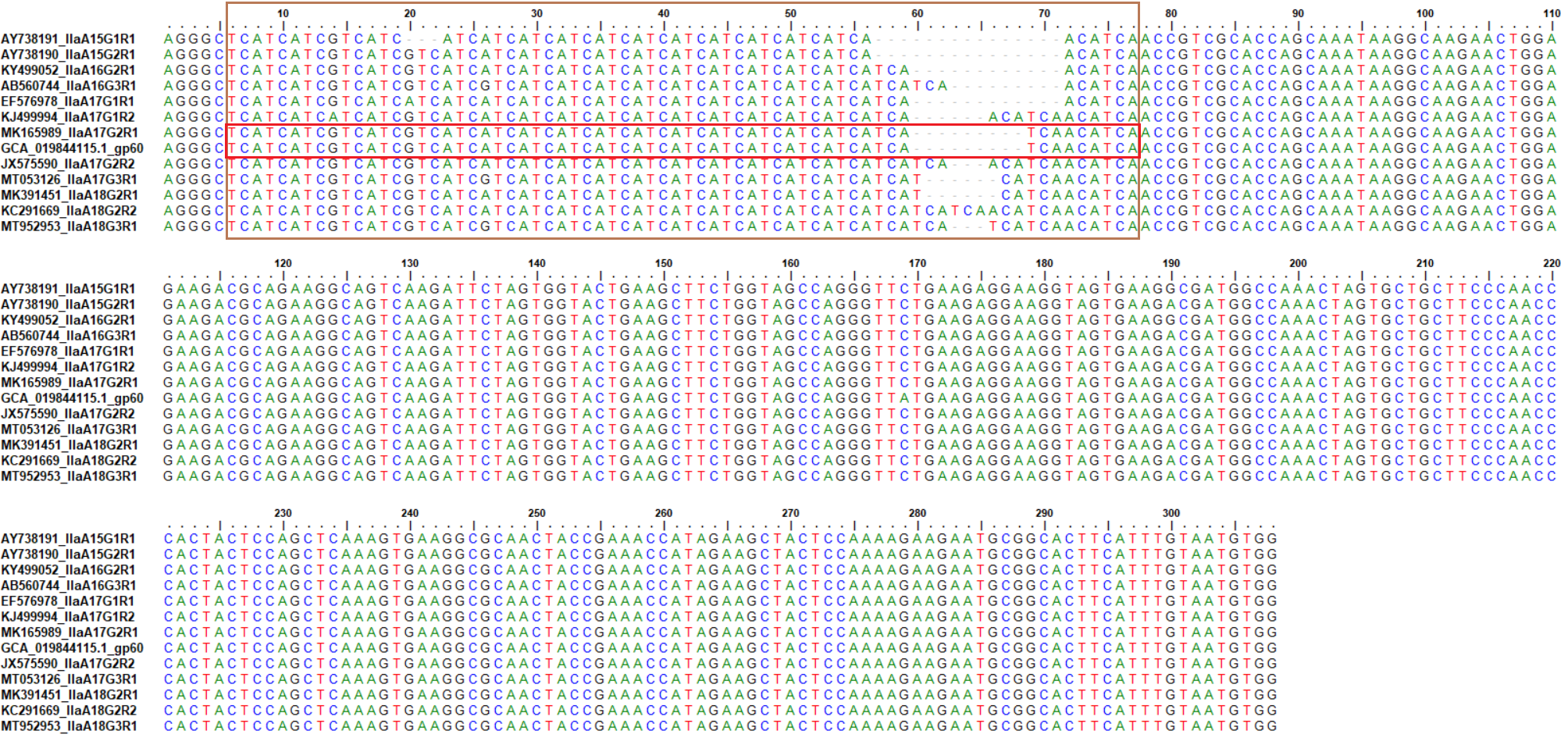


**Supplementary Figure 3**: Alignment of *gp60* subtyping region (indicated with a brown box) with the assembled genome. The genome assembly from the study (GCA_019844115.1_gp60) aligns with the reference sequence of IIaA17G2R1 (indicated with a red box, within the subtyping region).
