# Supplementary material for: Fully resolved assembly of Cryptosporidium parvum
Source: Gigascience. 2022 Feb 15;11:giac010. doi: 10.1093/gigascience/giac010 (PMC8848321; doi:10.1093/gigascience/giac010)
Supplement: giac010_Response_to_Reviewer_Comments_Original_Submission [file giac010_response_to_reviewer_comments_original_submission.pdf]

Author's Response To Reviewer Comments

Close

We have addressed all comments from the reviewers and have a detailed point to point response attached as personal cover letter. We would again like to thank the editor and the reviewers for their constructive feedback.

Thank you
Fritz Sedlazeck

Close
