# Supplementary material for: Fully resolved assembly of Cryptosporidium parvum
Source: Gigascience. 2022 Feb 15;11:giac010. doi: 10.1093/gigascience/giac010 (PMC8848321; doi:10.1093/gigascience/giac010)
Supplement: giac010_Reviewer_2_Report_Original_Submission — Matthew Knox -- 11/2/2021 Reviewed [file giac010_reviewer_2_report_original_submission.pdf]

Reviewer Report

Title: Fully resolved assembly of Cryptosporidium parvum

Version: Original Submission **Date:** 11/2/2021

Reviewer name: Matthew Knox

Reviewer Comments to Author:

Manuscript Number: GIGA-D-21-00321
Fully resolved assembly of Cryptosporidium parvum
Reviewer comments to authors.
Overall, Menon et al. present a significant contribution to the field with this work. Their fully resolved assembly of Cryptosporidium parvum is the first to my knowledge to utilize long read sequencing in whole genome sequencing for this group of protozoan parasites and as such provides validation of previously published work while also improving on current reference standards and providing a robust and well described analysis pipeline for future studies.
In my view, there are only a couple of issues with the paper that should be addressed. The first is a discussion of recent work using metabarcoding (e.g. DOI10.1016/j.meegid.2012.08.017, DOI10.1016/j.ijpara.2017.03.003), which demonstrates mixed infections in clinical samples of patients infected with Cryptosporidium which were missed with consensus Sanger sequencing. In some cases, mixtures of subtype families can be found, though dominance of a single subtype with a few closely related variants is more common and more likely in the current paper. Nonetheless, this may have implications for sequencing since purity of the "culture" cannot be guaranteed and results from the lack of reliable in vitro culture methods for Cryptosporidium.
The second issue I have is with the section on comparative genomics. Strictly speaking calling this a comparative genomics analysis is not correct since the authors do not compare genomes with genomes. Instead, it is based on comparison with a small subset of sanger generated sequences and does not add much to the paper in my view. If it is to be included, the text should be rephrased to better reflect the analyses and the identity (species, subtype, subtype family) of the sequences downloaded from genbank should be presented in more detail. Also, it is unclear what criteria were used to select these sequences from among the many hundreds available for C. parvum and this should be stated too.
In addition to significant comments above, I detected a few inconsistencies and typographical errors in the submission and have included minor comments (sticky notes) in the attached pdf document. I hope that the authors find this helpful in improving the manuscript.

Level of Interest

Please indicate how interesting you found the manuscript: Choose an item.

Quality of Written English

Please indicate the quality of language in the manuscript: Choose an item.

Declaration of Competing Interests

Please complete a declaration of competing interests, considering the following questions:

- Have you in the past five years received reimbursements, fees, funding, or salary from an organisation that may in any way gain or lose financially from the publication of this manuscript, either now or in the future?
- Do you hold any stocks or shares in an organisation that may in any way gain or lose financially from the publication of this manuscript, either now or in the future?
- Do you hold or are you currently applying for any patents relating to the content of the manuscript?
- Have you received reimbursements, fees, funding, or salary from an organization that holds or has applied for patents relating to the content of the manuscript?
- Do you have any other financial competing interests?
- Do you have any non-financial competing interests in relation to this paper?

If you can answer no to all of the above, write 'I declare that I have no competing interests' below. If your reply is yes to any, please give details below.

I declare that I have no competing interests

I agree to the open peer review policy of the journal. I understand that my name will be included on my report to the authors and, if the manuscript is accepted for publication, my named report including any attachments I upload will be posted on the website along with the authors' responses. I agree for my report to be made available under an Open Access Creative Commons CC-BY license (http://creativecommons.org/licenses/by/4.0/). I understand that any comments which I do not wish to be included in my named report can be included as confidential comments to the editors, which will not be published.

Choose an item.

To further support our reviewers, we have joined with Publons, where you can gain additional credit to further highlight your hard work (see: https://publons.com/journal/530/gigascience). On publication of this paper, your review will be automatically added to Publons, you can then choose whether or not to claim your Publons credit. I understand this statement.

Yes Choose an item.
