# Supplementary material for: Fully resolved assembly of Cryptosporidium parvum
Source: Gigascience. 2022 Feb 15;11:giac010. doi: 10.1093/gigascience/giac010 (PMC8848321; doi:10.1093/gigascience/giac010)
Supplement: giac010_Reviewer_3_Report_Original_Submission — Juan Alzate -- 11/2/2021 Reviewed [file giac010_reviewer_3_report_original_submission.pdf]

Reviewer Report

Title: Fully resolved assembly of Cryptosporidium parvum

Version: Original Submission **Date:** 11/2/2021

Reviewer name: Juan Alzate

Reviewer Comments to Author:

The present paper entitled "Fully resolved assembly of Cryptosporidium parvum" shows the results of the genomic sequencing of the protozoan parasite C. parvum using both 2nd (Novaseq) and 3rd (ONT) generations NGS technologies. Additionally, they assembled the C. parvum genome and compared their results with the previous C. parvum IOWAA II reference. The authors also undertake some QC analysis to validate chromosome models.
The paper is interesting because there is a need to have a fully resolved Cryptospodium genome. The sequencing by itself is not much an achievement, the authors applied commercially available platforms. In the assembly process, they also used already known assemblers and mapper tools.
I think BUSCO does not deliver the detailed results expected here. Maybe a more comprehensive analysis, including all the single-copy genes present in the C. parvum, can help to better support the quality of the genome.
One additional recommendation is that the authors present a detailed analysis of single nucleotide variants (SNVs). This data can be extracted from the same BAM files that the authors already generated for Structural Variants analysis. This analysis is particularly important because it can show the readers how clonal is the C. parvum strain used.
I don't know if this is possible. Can you compare your genome model with the one published here BioRxiv - DOI: 10.1101/2021.01.29.428682.?
Please make public the raw-read data. (Novaseq and ONT raw reads)
Please explain in more detail in the Methods section how do you find and analyze the structural variants.
I don't understand why to estimate the genome size. Could you explain it?

Level of Interest

Please indicate how interesting you found the manuscript: Choose an item.

Quality of Written English

Please indicate the quality of language in the manuscript: Choose an item.

Declaration of Competing Interests

Please complete a declaration of competing interests, considering the following questions:

- Have you in the past five years received reimbursements, fees, funding, or salary from an organisation that may in any way gain or lose financially from the publication of this manuscript, either now or in the future?
- Do you hold any stocks or shares in an organisation that may in any way gain or lose financially from the publication of this manuscript, either now or in the future?
- Do you hold or are you currently applying for any patents relating to the content of the manuscript?
- Have you received reimbursements, fees, funding, or salary from an organization that holds or has applied for patents relating to the content of the manuscript?
- Do you have any other financial competing interests?
- Do you have any non-financial competing interests in relation to this paper?

If you can answer no to all of the above, write 'I declare that I have no competing interests' below. If your reply is yes to any, please give details below.

I declare that I have no competing interests

I agree to the open peer review policy of the journal. I understand that my name will be included on my report to the authors and, if the manuscript is accepted for publication, my named report including any attachments I upload will be posted on the website along with the authors' responses. I agree for my report to be made available under an Open Access Creative Commons CC-BY license (http://creativecommons.org/licenses/by/4.0/). I understand that any comments which I do not wish to be included in my named report can be included as confidential comments to the editors, which will not be published.

Choose an item.

To further support our reviewers, we have joined with Publons, where you can gain additional credit to further highlight your hard work (see: https://publons.com/journal/530/gigascience). On publication of this paper, your review will be automatically added to Publons, you can then choose whether or not to claim your Publons credit. I understand this statement.

Yes Choose an item.
